# Lipid metabolism indicators provide tools for the diagnosis of non-alcoholic fatty liver disease: results of a nationwide survey

**DOI:** 10.3389/fendo.2024.1468228

**Published:** 2025-01-17

**Authors:** Yongxin Wang, Chang Fu, Hengwei Jin, Yibo Yang, Xiaocong Li, Kai Liu

**Affiliations:** ^1^ Department of Hepatobiliary and Pancreatic Surgery, General Surgery Center, The First Hospital of Jilin University, Changchun, Jilin, China; ^2^ Department of Pharmacy, China-Japan Friendship Hospital, Beijing, China; ^3^ Clinical Trial Research Center, China-Japan Friendship Hospital, Beijing, China

**Keywords:** visceral adiposity index, cardiometabolic index, lipid accumulation product, non-alcoholic fatty liver disease, cardiometabolic disease, NHANES

## Abstract

**Background:**

Cardiometabolic index (CMI), visceral adiposity index (VAI), and lipid accumulation product (LAP) are lipid-related parameters that reflect central obesity, which is closely associated with the development of non-alcoholic fatty liver disease (NAFLD). The aim of this study is to investigate the effectiveness of these lipid-related parameters in diagnosing NAFLD and to compare their predictive abilities.

**Methods:**

This population-based study extracted datasets from the National Health and Nutrition Examination Survey (NHANES) 2017–2020. CMI, VAI, and LAP were included in the multivariate logistic model as both continuous and categorical variables to assess the relationship between different lipid-related parameters and NAFLD. To further elucidate this connection, we utilized restricted cubic splines and conducted subgroup analysis. Additionally, the receiver operating characteristics (ROC) curve was employed to evaluate the predictive effectiveness of CMI, VAI, and LAP for NAFLD.

**Results:**

The study included 2,878 adults as the study population, of whom 1,263 participants were diagnosed with NAFLD. When lipid-related parameters were analyzed as continuous variables, they showed a positive correlation with NAFLD. The OR(95%CI) were 2.29(1.81,2.89) for CMI (per 1-unit), 1.40(1.28,1.52) for VAI (per 1-unit) and 1.15(1.11,1.20) for LAP (per 10-units). This correlation remains statistically significant when the lipid-related parameters are analyzed as categorical variables. In descending order of diagnostic capability for NAFLD, the AUC values are as follows: LAP (0.794), CMI (0.752), and VAI (0.719).

**Conclusion:**

CMI, VAI, and LAP may be important clinical indicators for identifying NAFLD, with LAP demonstrating the best predictive ability among them.

## Introduction

Non-alcoholic fatty liver disease (NAFLD) is a chronic liver condition marked by excessive lipid accumulation in hepatocytes except alcohol and other definite liver injury factors. NAFLD can progress from hepatic steatosis to non-alcoholic steatohepatitis (NASH) and, in more severe cases, advance to liver fibrosis and cirrhosis, ultimately leading to hepatocellular carcinoma (HCC) (1). As the most prevalent chronic liver disease, the number of NAFLD patients in America is projected to reach 100.9 million by 2030 (2). The overall prevalence of NAFLD was estimated to be 32.4% worldwide, and its incidence and liver-related mortality are increasing significantly (3, 4). NAFLD is a metabolic disease closely related to obesity, dyslipidemia, diabetes, hypertension and other metabolic disorders, and its etiology is still unclear (5, 6). To accurately reflect the primary drivers of the disease, the concept of metabolic dysfunction-associated fatty liver disease (MAFLD) has been proposed in recent years (7). NAFLD has greatly increased the burden of human health and social health care, so it is important to find effective clinical indicators to identify NAFLD as early as possible and reduce its risk.

Previous studies have indicated that visceral fat and dyslipidemia are significant risk factors for the development of NAFLD (8–12). As a composite index of waist circumference (WC), body mass index (BMI), triglyceride (TG) and high-density lipoprotein cholesterol (HDL-C), visceral adiposity index (VAI) can effectively reflect visceral fat accumulation and dysfunction (13). Xu et al.’s prospective cohort study based on the Chinese population and Okamura et al.’s longitudinal study based on the Japanese population both found that VAI can serve as a predictive indicator for NAFLD (14, 15). Cardiometabolic index (CMI) is a novel index that combines waist-to-height ratio and TG/HDL-C, and it is considered an effective measure for assessing visceral adipose tissue (16, 17). A study involving 14,251 Japanese individuals found that elevated CMI is independently linked to a higher risk of NAFLD (18). The lipid accumulation product (LAP), calculated using WC and TG, can more accurately reflect the degree of lipid accumulation (19). Although studies have shown that CMI, VAI, and LAP are associated with the risk of NAFLD, the predictive abilities of these lipid-related parameters for NAFLD have not yet been clarified.

Using the latest data from the nationally representative National Health and Nutrition Examination Survey (NHANES) database, this study aimed to compare the potential value of lipid-related parameters in predicting NAFLD among the adults in the U.S.

## Materials and methods

### Study design

NHANES formed representative population data in the United States by surveying different populations, which adopted a stratified multi-stage sampling design and was approved by the National Center for Health Statistics Ethics Review Board. The study followed the Declaration of Helsinki and obtained written informed consent from the study population.

**Figure 1:**
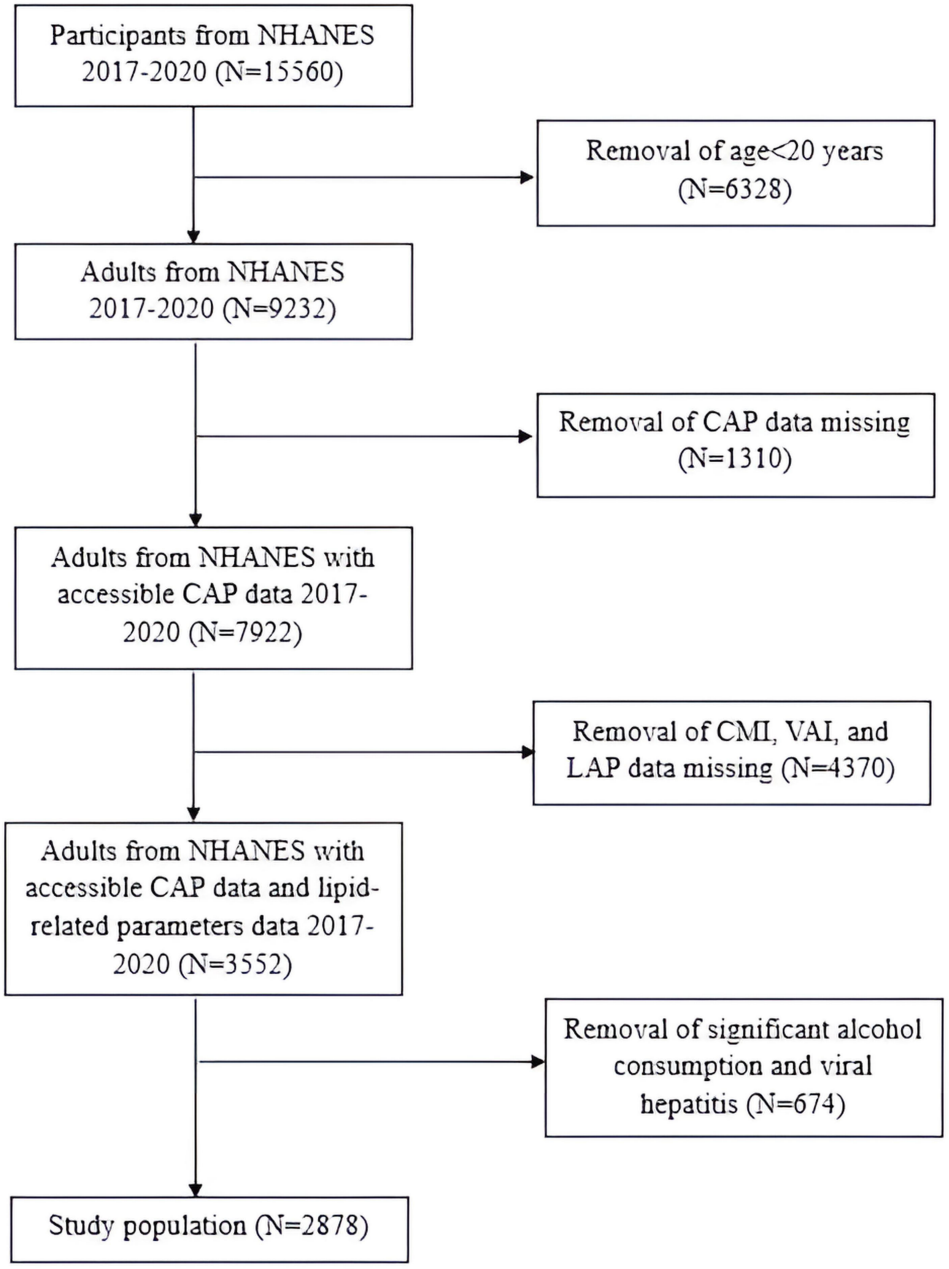
Flowchart of the study population.

In this research, we focused on the NHANES 2017-March 2020 cycle, which included 15,560 participants. The exclusion criteria for the study population were as follows (1): individuals aged< 20 years old; (2) individuals without controlled attenuation parameter (CAP) values; (3) individuals without WC, BMI, TG or HDL-C; (4) individuals who were positive for Hepatitis B virus (HBV) surface antigen or Hepatitis C virus (HCV) RNA; (5) individuals who drank more than 2 alcoholic beverages per day for females and more than 3 alcoholic beverages per day for males. Consequently, the final study population consisted of 2,878 participants (**Figure 1**).

### Study variables

CMI, VAI and LAP were calculated as follows:


$$CMI=\frac{WC}{Height}\times \frac{TG}{HDL-C}$$

$$\begin{array}{c} VAI=\{WC/[39.68+\left(1.88\times BMI\right)]\}\;\times \left(TG/1.03\right)\\ \times \left(1.31/HDL-C\right) \end{array}$$

for males;


$$\begin{array}{c} VAI=\{WC/[36.58+\left(1.89\times BMI\right)]\}\;\times \left(TG/0.81\right)\\ \times \left(1.52/HDL-C\right) \end{array}$$

for females;


LAP=(WC−65)×TG 

for males;


LAP=(WC−58)×TG

for females

WC and height were measured in cm, BMI was reported in kg/m², TG and HDL-C were measured in mmol/L. The diagnosis of NAFLD was based on the CAP value obtained by liver ultrasound transient elastography, and the diagnostic criterion was CAP≥274 dB/m (20).

Covariates included age, gender, race, BMI, education level, marital status, poverty income ratio (PIR), WC, HDL-C, TG, diabetes, hypertension, smoking and drinking status. Education level was categorized into three groups: below high school, high school, and above high school. Smoking status was determined by asking participants if they had smoked at least 100 cigarettes in their lifetime. Drinking status was assessed by asking participants if they had consumed at least 12 alcoholic drinks in one year.

All study variables were obtained from the publicly available NHANES dataset. These variables were selected based on their relevance to the study objectives.

### Statistical analysis

Continuous variables conforming to a normal distribution were presented as mean ± standard deviation (SD). Conversely, those not following a normal distribution were depicted by the median along with the interquartile range (IQR). Categorical variables were reported as frequencies (n) and proportions (%). To analyze continuous variables, ANOVA and Kruskal-Wallis H tests were employed, while chi-square tests were used to compare categorical variables. This analysis aimed to investigate the characteristics of the participants with and without NAFLD. Study population was categorized into quartiles according to their CMI, VAI, and LAP values, designated as Q1, Q2, Q3, and Q4. To assess the correlation between lipid-related parameters and NAFLD, multivariate logistic regression was applied across three models. The strength of these associations was expressed using odds ratios (OR) and 95% confidence intervals (CI). Model 1 made no adjustments for covariates. Model 2 adjusted for gender, age, and race, while Model 3 accounted for all covariates. At first, lipid-related parameters were included in logistic models as continuous variables and then categorized into quartiles. The potential nonlinear dose-response relationships between these lipid-related parameters and NAFLD were investigated by restricted cubic splines (RCS). Additionally, we conducted a subgroup analysis to assess whether this association varies among different populations. To compare the predictive performance of the lipid-related parameters for NAFLD, we calculated the area under the curve (AUC) for each parameter and explored their respective cutoff values. All analyses were performed using SAS version 9.4, with a two-sided P-value of less than 0.05 considered statistically significant.

## Results

### Characteristics of the study population

The study included 2,878 participants, comprising 1,374 males and 1,504 females. According to CAP, 1,263 participants were diagnosed with NAFLD, resulting in a prevalence rate of 43.9%. The mean ± SD values for the lipid-related parameters (CMI, VAI, and LAP) were 0.7 ± 1.0, 1.8 ± 2.2 and 51.7 ± 56.8. We described the characteristics of the study population based on the presence or absence of NAFLD (**Table 1**). Generally, individuals with NAFLD tend to be older, more frequently male, and show a higher prevalence of diabetes and hypertension. As components of lipid-related parameters, BMI, WC and TG were significantly increased in participants with NAFLD, while HDL-C was significantly decreased. Furthermore, participants with NAFLD demonstrated significantly higher levels of CMI, VAI, and LAP compared to those without the disease (P<0.0001).

**Table 1:** Comparison of characteristics between participants with and without NAFLD.

Characteristics	Total (N=2878)	Without NAFLD (N=1615)	With NAFLD (N=1263)	P Value
Age (year), mean ± SD	52.3 ± 16.9	50.1 ± 17.8	55.1 ± 15.3	<0.0001
Gender, n (%)				<0.0001
Male	1374 (47.7)	718 (44.5)	656 (51.9)	
Female	1504 (52.3)	897 (55.5)	607 (48.1)	
Race, n (%)				<0.0001
Mexican American	333 (11.6)	145 (9.0)	188 (14.9)	
Other Hispanic	297 (10.3)	166 (10.3)	131 (10.4)	
Non-Hispanic White	971 (33.7)	510 (31.6)	461 (36.5)	
Non-Hispanic Black	732 (25.4)	468 (29.0)	264 (20.9)	
Other Race	545 (18.9)	326 (20.2)	219 (17.3)	
Education level, n (%)				0.1830
Less than high school	526 (18.3)	279 (17.3)	247 (19.6)	
High school	659 (22.9)	364 (22.6)	295 (23.4)	
More than high school	1691 (58.8)	971 (60.2)	720 (57.1)	
Marital status, n (%)				<0.0001
Cohabitation	1758 (61.2)	924 (57.3)	834 (66.1)	
Solitude	1116 (38.8)	689 (42.7)	427 (33.9)	
PIR, mean ± SD	2.7 ± 1.6	2.7 ± 1.6	2.7 ± 1.6	0.4812
Smoking status, n (%)	1127 (39.2)	612 (37.9)	515 (40.8)	0.1223
Drinking status, n (%)	1078 (44.1)	641 (47.0)	437 (40.4)	0.0012
Diabetes, n (%)	477 (16.6)	158 (9.8)	319 (25.3)	<0.0001
Hypertension, n (%)	1138 (39.6)	506 (31.4)	632 (50.1)	<0.0001
BMI (kg/m^2^), mean ± SD	29.7 ± 7.1	27.0 ± 5.9	33.2 ± 7.1	<0.0001
Height (cm), mean ± SD	166.4 ± 10.0	166.0 ± 10.0	167.0 ± 10.1	0.0059
WC (cm), mean ± SD	100.5 ± 16.8	93.3 ± 14.2	109.7 ± 15.4	<0.0001
TG (mmol/L), mean ± SD	1.2 ± 1.1	1.0 ± 0.8	1.5 ± 1.4	<0.0001
HDL-C (mmol/L), mean ± SD	1.4 ± 0.4	1.5 ± 0.4	1.3 ± 0.4	<0.0001
CMI, mean ± SD	0.7 ± 1.0	0.5 ± 0.6	0.9 ± 1.2	<0.0001
VAI, mean ± SD	1.8 ± 2.2	1.3 ± 1.4	2.4 ± 2.8	<0.0001
LAP, mean ± SD	51.7 ± 56.8	34.1 ± 32.7	74.1 ± 71.4	<0.0001

*NAFLD, non-alcoholic fatty liver disease; PIR, poverty income ratio; BMI, body mass index; HDL-C, high-density lipoprotein cholesterol; WC, waist circumference; TG, triglyceride; CMI, cardiometabolic index; VAI, visceral adiposity index; LAP, lipid accumulation product.

**Table 2** presents the characteristics of the participants grouped according to the quartiles of CMI, VAI, and LAP. In the groups based on different indicators, it was found that the subgroups with higher lipid-related parameters had a higher proportion of people with NAFLD, diabetes, and hypertension. Additionally, BMI, WC, and TG were higher in groups with higher lipid-related parameters, while HDL-C was lower in these groups.

**Table 2:** Comparison of intergroup differences based on grouping by lipid-related parameters.

Characteristics	CMI				VAI				LAP			
	Q1 (<0.26) (N=719)	Q2 (0.26-0.46) (N=720)	Q3 (0.46-0.81) (N=719)	Q4 (>0.81) (N=720)	Q1 (<0.75) (N=719)	Q2 (0.75-1.27) (N=720)	Q3 (1.27-2.15) (N=719)	Q4 (>2.15) (N=720)	Q1(<0.75) (N=719)	Q2 (20.68-38.76) (N=720)	Q3 (38.76-65.18) (N=719)	Q4 (>65.18) (N=720)
NAFLD, n (%)	122 (17.0)	234 (32.5)	391 (54.4)	516 (71.7)	154 (21.4)	242 (33.6)	387 (53.8)	480 (66.7)	86 (12.0)	228 (31.7)	391 (54.4)	558 (77.5)
Age (year), mean ± SD	47.6 ± 18.0	52.5 ± 17.1	54.4 ± 16.1	54.6 ± 15.5	48.1 ± 18.1	51.9 ± 17.0	54.5 ± 16.3	54.6 ± 15.4	44.6 ± 17.5	53.7 ± 16.9	55.2 ± 15.6	55.6 ± 15.3
Gender, n (%)												
Male	289 (40.2)	337 (46.8)	340 (47.3)	408 (56.7)	376 (52.3)	348 (48.3)	332 (46.2)	318 (44.2)	351 (48.8)	340 (47.2)	341 (47.4)	342 (47.5)
Female	430 (59.8)	383 (53.2)	379 (52.7)	312 (43.3)	343 (47.7)	372 (51.7)	387 (53.8)	402 (55.8)	368 (51.2)	380 (52.8)	378 (52.6)	378 (52.5)
Race, n (%)												
Mexican American	51 (7.1)	87 (12.1)	80 (11.1)	115 (16.0)	55 (7.6)	84 (11.7)	83 (11.5)	111 (15.4)	52 (7.2)	91 (12.6)	85 (11.8)	105 (14.6)
Other Hispanic	48 (6.7)	68 (9.4)	92 (12.8)	89 (12.4)	46 (6.4)	78 (10.8)	78 (10.8)	95 (13.2)	50 (7.0)	74 (10.3)	88 (12.2)	85 (11.8)
Non-Hispanic White	227 (31.6)	225 (31.3)	225 (31.3)	294 (40.8)	219 (30.5)	216 (30.0)	245 (34.1)	291 (40.4)	208 (28.9)	215 (29.9)	231 (32.1)	317 (44.0)
Non-Hispanic Black	253 (35.2)	219 (30.4)	181 (25.2)	79 (11.0)	265 (36.9)	222 (30.8)	171 (23.8)	74 (10.3)	239 (33.2)	196 (27.2)	196 (27.3)	101 (14.0)
Other Race	140 (19.5)	121 (16.8)	141 (19.6)	143 (19.9)	134 (18.6)	120 (16.7)	142 (19.7)	149 (20.7)	170 (23.6)	144 (20.0)	119 (16.6)	112 (15.6)
Education level, n (%)												
Less than high school	81 (11.3)	114 (15.8)	155 (21.6)	176 (24.4)	94 (13.1)	100 (13.9)	155 (21.6)	177 (24.6)	87 (12.1)	130 (18.1)	145 (20.2)	164 (22.8)
High school	167 (23.3)	180 (25.0)	154 (21.4)	158 (21.9)	168 (23.4)	175 (24.3)	165 (22.9)	151 (21.0)	184 (25.6)	164 (22.8)	146 (20.3)	165 (22.9)
More than high school	469 (65.4)	426 (59.2)	410 (57.0)	386 (53.6)	455 (63.5)	445 (61.8)	399 (55.5)	392 (54.4)	447 (62.3)	425 (59.1)	428 (59.5)	391 (54.3)
Marital status, n (%)												
Cohabitation	398 (55.6)	436 (60.6)	454 (63.1)	470 (65.4)	405 (56.5)	439 (61.1)	450 (62.6)	464 (64.5)	402 (56.0)	447 (62.3)	456 (63.4)	453 (63.0)
Solitude	318 (44.4)	284 (39.4)	265 (36.9)	249 (34.6)	312 (43.5)	280 (38.9)	269 (37.4)	255 (35.5)	316 (44.0)	271 (37.7)	263 (36.6)	266 (37.0)
PIR, mean ± SD	2.9 ± 1.6	2.7 ± 1.6	2.6 ± 1.6	2.6 ± 1.6	2.9 ± 1.6	2.8 ± 1.6	2.6 ± 1.6	2.6 ± 1.6	2.8 ± 1.6	2.8 ± 1.6	2.7 ± 1.6	2.6 ± 1.6
Smoking, n (%)	251 (34.9)	267 (37.2)	280 (38.9)	329 (45.7)	265 (36.9)	255 (35.5)	298 (41.4)	309 (42.9)	254 (35.4)	263 (36.5)	279 (38.9)	331 (46.0)
Drinking, n (%)	329 (53.8)	273 (45.2)	244 (39.2)	232 (38.2)	337 (54.4)	289 (47.4)	236 (38.2)	216 (36.1)	312 (51.6)	284 (47.2)	258 (41.3)	224 (36.5)
Diabetes, n (%)	45 (6.3)	96 (13.4)	137 (19.1)	199 (27.6)	59 (8.2)	90 (12.5)	135 (18.8)	193 (26.8)	49 (6.8)	79 (11.0)	143 (19.9)	206 (28.6)
Hypertension, n (%)	192 (26.7)	267 (37.1)	321 (44.8)	358 (49.7)	208 (28.9)	254 (35.3)	327 (45.5)	349 (48.6)	149 (20.7)	267 (37.1)	331 (46.2)	391 (54.3)
BMI (kg/m^2^), mean ± SD	25.2 ± 5.1	29.0 ± 6.3	31.2 ± 6.9	33.4 ± 7.3	26.3 ± 5.9	29.2 ± 7.1	31.1 ± 7.1	32.2 ± 6.9	23.7 ± 3.9	28.4 ± 4.9	31.2 ± 6.0	35.5 ± 7.4
Height (cm), mean ± SD	166.6 ± 9.8	167.1 ± 10.2	165.5 ± 10.0	166.5 ± 10.1	168.1 ± 10.0	166.9 ± 9.9	165.6 ± 10.2	165.1 ± 9.9	167.1 ± 9.9	166.2 ± 10.1	166.3 ± 10.1	166.1 ± 10.1
WC (cm), mean ± SD	87.5 ± 12.7	99.3 ± 14.8	104.3 ± 14.7	110.7 ± 15.8	90.5 ± 14.8	99.2 ± 16.0	104.7 ± 15.9	107.5 ± 15.4	83.5 ± 9.6	97.4 ± 10.9	105.5 ± 12.8	115.4 ± 14.7
TG (mmol/L), mean ± SD	0.6 ± 0.2	0.9 ± 0.2	1.2 ± 0.3	2.3 ± 1.8	0.6 ± 0.2	0.9 ± 0.2	1.2 ± 0.3	2.3 ± 1.8	0.6 ± 0.2	0.9 ± 0.3	1.2 ± 0.4	2.2 ± 1.8
HDL-C (mmol/L), mean ± SD	1.8 ± 0.4	1.4 ± 0.3	1.3 ± 0.2	1.1 ± 0.2	1.7 ± 0.4	1.4 ± 0.3	1.3 ± 0.2	1.1 ± 0.2	1.7 ± 0.4	1.4 ± 0.4	1.3 ± 0.3	1.1 ± 0.3
CMI	0.2 ± 0.1	0.4 ± 0.1	0.6 ± 0.1	1.6 ± 1.6	0.2 ± 0.1	0.4 ± 0.1	0.6 ± 0.2	1.5 ± 1.6	0.2 ± 0.1	0.4 ± 0.1	0.6 ± 0.2	1.5 ± 1.6
VAI	0.5 ± 0.2	1.0 ± 0.2	1.7 ± 0.4	3.8 ± 3.6	0.5 ± 0.1	1.0 ± 0.1	1.7 ± 0.2	3.9 ± 3.6	0.6 ± 0.3	1.1 ± 0.4	1.7 ± 0.7	3.7 ± 3.7
LAP	14.8 ± 7.7	31.6 ± 11.9	51.1 ± 16.6	109.1 ± 85.9	16.0 ± 9.3	31.8 ± 13.2	52.3 ± 20.1	106.6 ± 87.0	12.7 ± 5.0	29.6 ± 5.1	50.6 ± 7.5	113.6 ± 83.5

*NAFLD, non-alcoholic fatty liver disease; PIR, poverty income ratio; BMI, body mass index; HDL-C, high-density lipoprotein cholesterol; WC, waist circumference; TG, triglyceride; CMI, cardiometabolic index; VAI, visceral adiposity index; LAP, lipid accumulation product.

### The correlation between lipid-related parameters and NAFLD

**Table 3** illustrates the association between lipid-related parameters and NAFLD. Analyzing these parameters as continuous variables, a positive relationship with NAFLD is evident across all three models. In Model 3, each 1-unit increase in CMI is associated with a 1.29-fold increase in the risk of developing NAFLD [OR (95% CI): 2.29 (1.81, 2.89)]. Similarly, each 1-unit increase in VAI corresponds to a 40% higher risk of NAFLD [OR (95% CI): 1.40 (1.28, 1.52)]. Additionally, for every 10-unit rise in LAP, the risk of NAFLD increases by 15% [OR (95% CI): 1.15 (1.11, 1.20)]. The association between lipid-related parameters and NAFLD remains statistically significant when these parameters are included in the model as categorical variables. Participants with higher quartiles of lipid-related parameters have a greater risk of developing NAFLD.

**Table 3:** Association between CMI, VAI, LAP and NAFLD.

Exposure	Model 1 OR (95%CI)	Model 2 OR (95%CI)	Model 3 OR (95%CI)
CMI (per 1-unit)	5.43 (4.46,6.62)	4.96 (4.06,6.07)	2.29 (1.81,2.89)
CMI (quartile)			
Q1	1.00 (Reference)	1.00 (Reference)	1.00 (Reference)
Q2	2.36 (1.84,3.02)	2.17 (1.69,2.79)	1.27 (0.92,1.75)
Q3	5.83 (4.57,7.44)	5.41 (4.22,6.93)	2.53 (1.83,3.48)
Q4	12.38 (9.61,15.94)	11.14 (8.58,14.46)	3.85 (2.73,5.43)
VAI (per 1-unit)	1.70 (1.59,1.83)	1.68 (1.56,1.80)	1.40 (1.28,1.52)
VAI (quartile)			
Q1	1.00 (Reference)	1.00 (Reference)	1.00 (Reference)
Q2	1.86 (1.47,2.35)	1.79 (1.41,2.28)	1.36 (1.00,1.86)
Q3	4.28 (3.40,5.39)	4.14 (3.27,5.25)	2.33 (1.71,3.17)
Q4	7.34 (5.79,9.29)	7.13 (5.57,9.13)	3.57 (2.58,4.93)
LAP (per 10-unit)	1.35 (1.32,1.40)	1.34 (1.30,1.39)	1.15 (1.11,1.20)
LAP (quartile)			
Q1	1.00 (Reference)	1.00 (Reference)	1.00 (Reference)
Q2	3.41 (2.59,4.49)	3.20 (2.42,4.24)	2.00 (1.41,2.84)
Q3	8.77 (6.71,11.48)	8.40 (6.37,11.07)	3.59 (2.51,5.14)
Q4	25.35 (19.06,33.72)	24.28 (18.07,32.62)	6.51 (4.32,9.80)

Model 1: no covariates were adjusted;

Model 2: age, gender, and race were adjusted;

Model 3: age, gender, race, BMI, education level, marital status, PIR, diabetes, hypertension, smoking and drinking status were adjusted.

**Figure 2:**
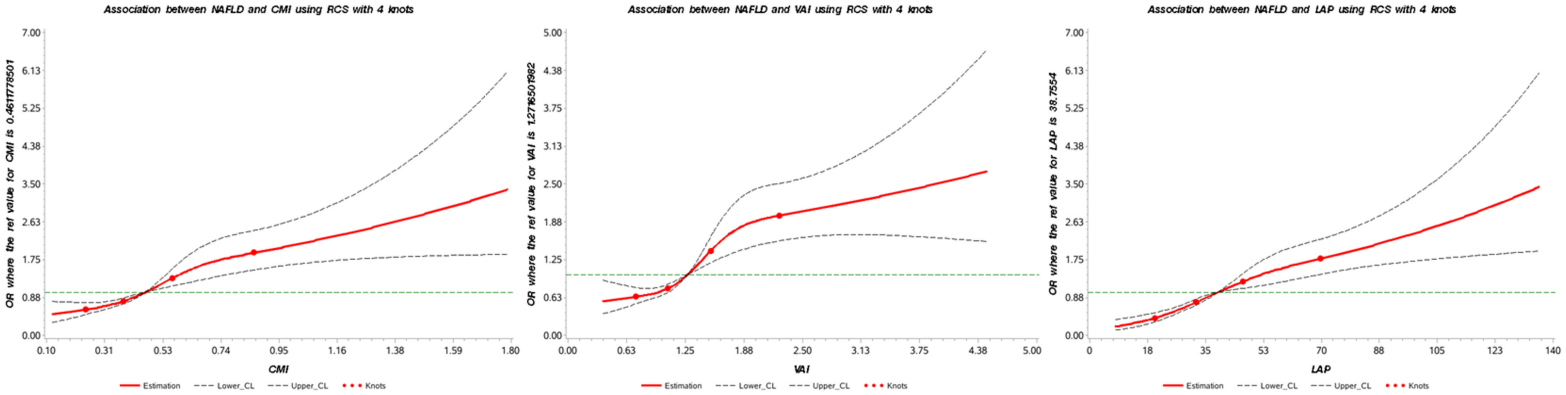
Association between lipid-related parameters and NAFLD. The red solid line represents the smoothed curve fit between the variables, while the black dashed lines indicate the 95% confidence interval of the fit. CMI, cardiometabolic index; VAI, visceral adiposity index; LAP, lipid accumulation product.

The RCS plot shown in **Figure 2** visualizes the association between lipid-related parameters and the prevalence of NAFLD. After adjusting for all confounding factors, an increased risk of NAFLD was observed with higher lipid-related parameters.

### Comparison of lipid-related parameters

**Figure 3:**
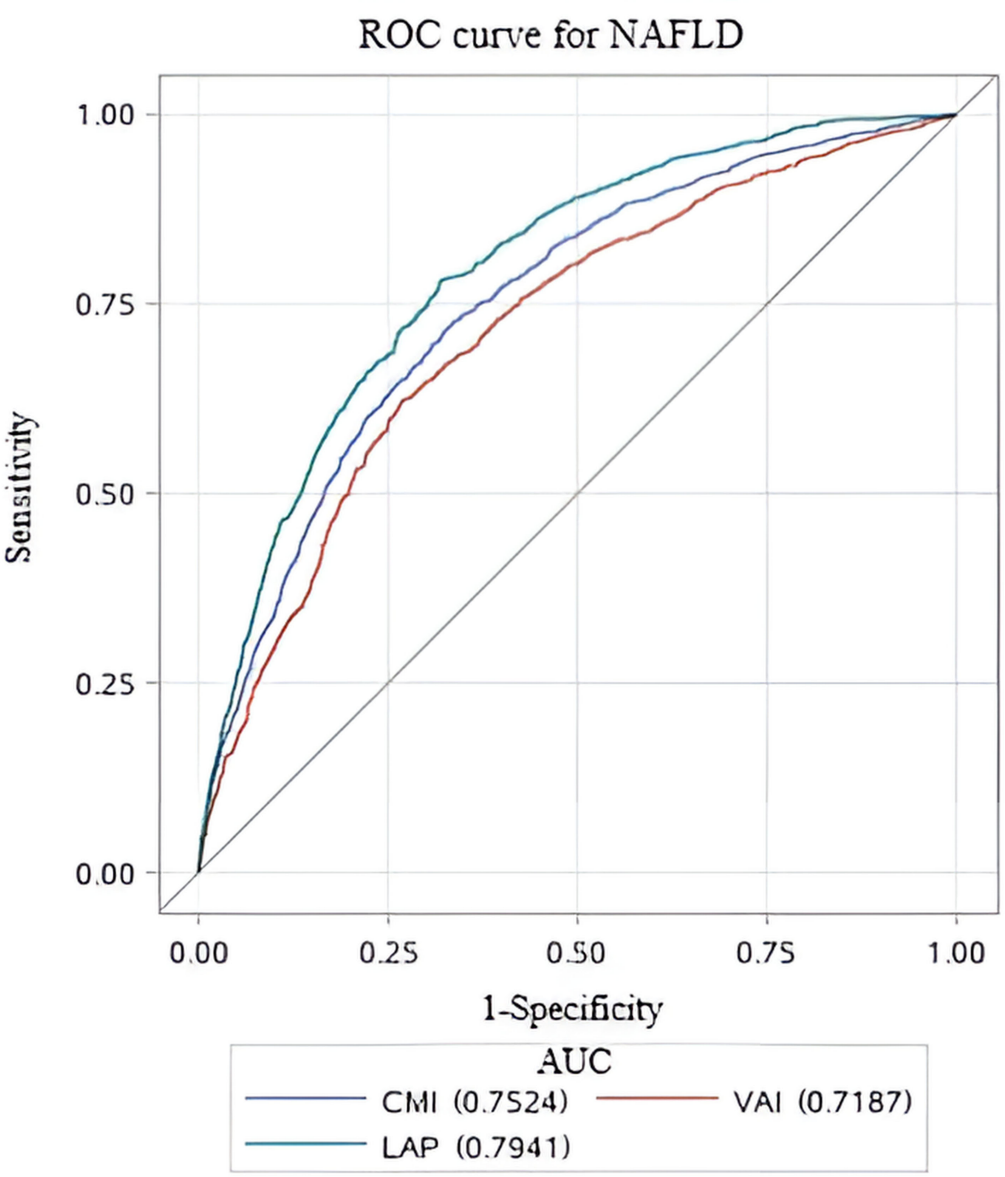
ROC curves for different lipid-related parameters to predict NAFLD. ROC, Receiver Operating Characteristic; AUC, area under the curve.

To assess the predictive performance of lipid-related parameters for NAFLD, receiver operating characteristics (ROC) curve was generated. Among these lipid-related parameters, LAP exhibited the highest predictive capability, with an AUC of 0.794 (95% CI: 0.778, 0.810). CMI came next, showing an AUC (95% CI) of 0.752 (0.735, 0.770). In contrast, VAI had comparatively weaker predictive power for NAFLD, with an AUC (95% CI) of 0.719 (0.700, 0.738). **Figure 3** displays the ROC curves. According to the principle of closest proximity to (0,1), the optimal cut-off values for CMI, VAI, and LAP should be set at 0.465, 1.341, and 37.02, respectively. At this point, the sensitivity of CMI, VAI, and LAP were 0.717, 0.671, and 0.781, respectively, while the specificity was 0.674, 0.676, and 0.682, respectively (**Table 4**).

**Table 4:** Evaluation of the performance of lipid-related parameters in predicting NAFLD.

Variables	AUC (95%CI)	Cutoff threshold	Sensitivity	Specificity
CMI	0.752 (0.735, 0.770)	0.465	0.717	0.674
VAI	0.719 (0.700, 0.738)	1.341	0.671	0.676
LAP	0.794 (0.778, 0.810)	37.02	0.781	0.682

### Subgroup analysis

**Figure 4:**
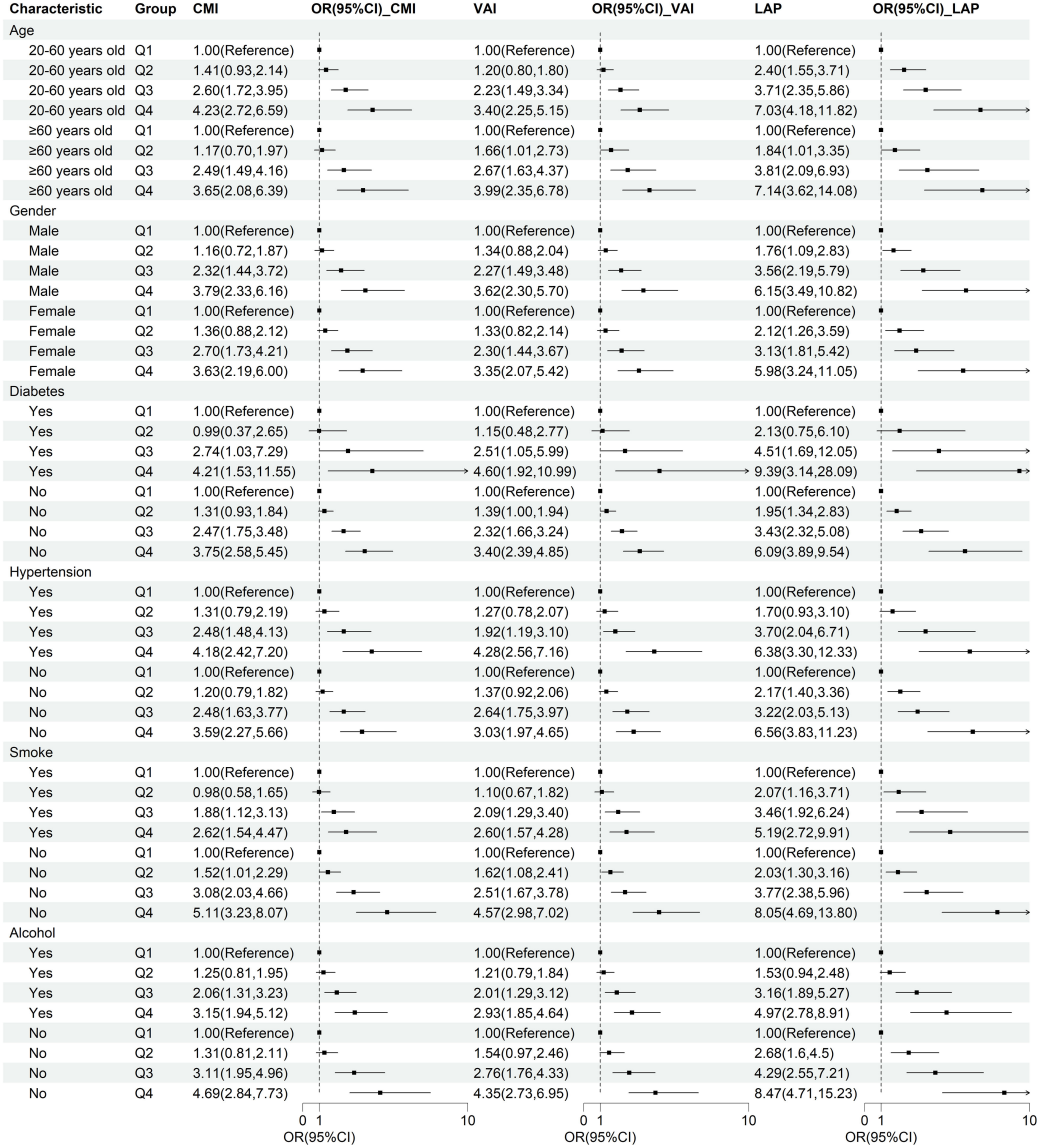
Subgroup analysis of the correlation between CMI, VAI, LAP, and NAFLD. Stratification factors include gender, age, smoking and drinking status, diabetes, and hypertension.

To verify the robustness of lipid-related parameters in predicting NAFLD risk in different populations, we further performed subgroup analysis. The results of the subgroup analysis demonstrated the robustness of the relationship between lipid-related parameters and NAFLD across different populations, with this association being more pronounced in individuals with diabetes, non-smokers, and non-drinkers (**Figure 4**).

## Discussion

In this large national survey, we confirmed the association between lipid-related parameters and NAFLD, and validated the reliability of the results across different subpopulations. This study also compared the predictive abilities of CMI, VAI, and LAP, with LAP demonstrating superior diagnostic capability.

Previous researches have explored the relationship between CMI, VAI, LAP, and NAFLD. A cross-sectional study of 7,238 participants found a positive association between VAI and the risk of NAFLD [OR (95%CI): 1.291(1.223,1.362)], and NAFLD patients had higher BMI, blood pressure, fasting blood glucose (FBG), TG and WC, and lower HDL-C levels (p< 0.05) (21). In addition, a meta-analysis of 24 studies confirmed the reliability of VAI for predicting NAFLD (AUC = 0.767) (22). The study conducted by Li et al., based on NHANES data, also confirmed the association between VAI and NAFLD among U.S. adults (23). A study conducted in the Chinese population suggested that LAP and CMI are convenient indicators for screening and quantifying NAFLD, with a stronger association observed in females (24). A recent study based on the U.S. population found that an increase of one unit in CMI is associated with a 44% increased risk of NAFLD [OR (95%CI): 1.44(1.44,1.45)] (25). Ebrahimi et al. conducted a meta-analysis to assess the diagnostic value of LAP for NAFLD, revealing a sensitivity of 94% and a specificity of 85% (26). Our study utilized the latest NHANES data to confirm the reliability of CMI, VAI, and LAP in predicting the risk of NAFLD among U.S. adults.

CMI, VAI, and LAP, as novel lipid-related parameters, can more effectively reflect the visceral adipose tissue (VAT) than the traditional obesity indicators. VAT may be involved in the pathophysiological mechanism of the occurrence and development of NAFLD through the following ways. Firstly, excessive accumulation of visceral adipose tissue releases free fatty acids (FFA) through lipid interpretation, which reaches the liver through the portal vein and becomes the main source of TG in the liver, and further promotes the development of hepatic steatosis (27–30). Secondly, accumulation of free fatty acids in the liver induces insulin resistance (IR) by inhibiting glucose transport or phosphorylation in muscle (31, 32). IR can not only directly cause NAFLD by enhancing *de novo* lipogenesis in the liver, but also indirectly promote NAFLD by reducing the inhibition of lipolysis in adipose tissue, leading to increased free fatty acid (FFA) delivery to the liver (33, 34). Thirdly, visceral adipose dysfunction disrupts normal metabolic function by increasing inflammatory adipokines, including interleukin-6 (IL-6), macrophage chemoattractant protein-1 (MCP-1), and tumor necrosis factor-α (TNF-α) (35–37). Moreover, oxidative stress resulting from visceral fat accumulation can lead to liver inflammation, NAFLD (38, 39).

The strength of this research lies in the nationally representative study population, which ensures that the predictive value of lipid-related parameters for NAFLD is broadly applicable to the U.S. adult population. In addition, adjustment for potential confounders and the performance of subgroup analyses ensured the reliability of our findings. Several limitations of this study should be acknowledged. In this study, the diagnosis of NAFLD was based on liver ultrasound transient elastography. Although previous studies have shown that its accuracy is very high, there is still a certain gap compared with liver biopsy (40, 41). What’s more, because of the design limitations of the survey, the influence of potential confounding factors, including diet and drug use, could not be ruled out.

## Conclusions

CMI, VAI, and LAP emerged as useful indicators for identifying NAFLD risk, with LAP showing the highest predictive ability among them in this study. As an easily obtainable clinical indicator, LAP may offer a more practical and cost-effective option for clinical application. However, further research is needed to validate these findings.

## Data Availability

Publicly available datasets were analyzed in this study. This data can be found here: https://www.cdc.gov/nchs/nhanes.

## References

[1] Rinella ME Neuschwander-Tetri BA Siddiqui MS Abdelmalek MF Caldwell S Barb D. AASLD Practice Guidance on the clinical assessment and management of nonalcoholic fatty liver disease. Hepatology. (2023) 77:1797–835. doi: 10.1097/HEP.0000000000000323 PMC10735173 36727674

[2] Friedman SL Neuschwander-Tetri BA Rinella M Sanyal AJ. Mechanisms of NAFLD development and therapeutic strategies. Nat Med. (2018) 24:908–22. doi: 10.1038/s41591-018-0104-9 PMC6553468 29967350

[3] Riazi K Azhari H Charette JH Underwood FE King JA Afshar EE. The prevalence and incidence of NAFLD worldwide: a systematic review and meta-analysis. Lancet Gastroenterol Hepatol. (2022) 7:851–61. doi: 10.1016/S2468-1253(22)00165-0 35798021

[4] Paik JM Henry L Younossi Y Ong J Alqahtani S Younossi ZM. The burden of nonalcoholic fatty liver disease (NAFLD) is rapidly growing in every region of the world from 1990 to 2019. Hepatol Commun. (2023) 7(10):e0251. doi: 10.1097/HC9.0000000000000251 37782469 PMC10545420

[5] Kim D Touros A Kim WR. Nonalcoholic fatty liver disease and metabolic syndrome. Clin Liver Dis. (2018) 22:133–40. doi: 10.1016/j.cld.2017.08.010 29128053

[6] Sun Z Miller RA Patel RT Chen J Dhir R Wang H. Hepatic Hdac3 promotes gluconeogenesis by repressing lipid synthesis and sequestration. Nat Med. (2012) 18:934–42. doi: 10.1038/nm.2744 PMC3411870 22561686

[7] Eslam M Sanyal AJ George J. MAFLD: A consensus-driven proposed nomenclature for metabolic associated fatty liver disease. Gastroenterology. (2020) 158:1999–2014.e1. doi: 10.1053/j.gastro.2019.11.312 32044314

[8] Eguchi Y Eguchi T Mizuta T Ide Y Yasutake T Iwakiri R. Visceral fat accumulation and insulin resistance are important factors in nonalcoholic fatty liver disease. J Gastroenterol. (2006) 41:462–9. doi: 10.1007/s00535-006-1790-5 16799888

[9] Koda M Kawakami M Murawaki Y Senda M. The impact of visceral fat in nonalcoholic fatty liver disease: cross-sectional and longitudinal studies. J Gastroenterol. (2007) 42:897–903. doi: 10.1007/s00535-007-2107-z 18008034

[10] Saponaro C Sabatini S Gaggini M Carli F Rosso C Positano V. Adipose tissue dysfunction and visceral fat are associated with hepatic insulin resistance and severity of NASH even in lean individuals. Liver Int. (2022) 42:2418–27. doi: 10.1111/liv.v42.11 35900229

[11] Amor AJ Perea V. Dyslipidemia in nonalcoholic fatty liver disease. Curr Opin Endocrinol Diabetes Obes. (2019) 26:103–8. doi: 10.1097/MED.0000000000000464 30694825

[12] Liu Q Bengmark S Qu S. The role of hepatic fat accumulation in pathogenesis of non-alcoholic fatty liver disease (NAFLD). Lipids Health Dis. (2010) 9:42. doi: 10.1186/1476-511X-9-42 20426802 PMC2873482

[13] Amato MC Giordano C Galia M Criscimanna A Vitabile S Midiri M. Visceral Adiposity Index: a reliable indicator of visceral fat function associated with cardiometabolic risk. Diabetes Care. (2010) 33:920–2. doi: 10.2337/dc09-1825 PMC2845052 20067971

[14] Xu C Ma Z Wang Y Liu X Tao L Zheng D. Visceral adiposity index as a predictor of NAFLD: A prospective study with 4-year follow-up. Liver Int. (2018) 38:2294–300. doi: 10.1111/liv.2018.38.issue-12 30099825

[15] Okamura T Hashimoto Y Hamaguchi M Obora A Kojima T Fukui M. The visceral adiposity index is a predictor of incident nonalcoholic fatty liver disease: A population-based longitudinal study. Clin Res Hepatol Gastroenterol. (2020) 44:375–83. doi: 10.1016/j.clinre.2019.04.002 32434704

[16] Wakabayashi I Daimon T. The “cardiometabolic index” as a new marker determined by adiposity and blood lipids for discrimination of diabetes mellitus. Clin Chim Acta. (2015) 438:274–8. doi: 10.1016/j.cca.2014.08.042 25199852

[17] Wang H Sun Y Wang S Qian H Jia P Chen Y. Body adiposity index, lipid accumulation product, and cardiometabolic index reveal the contribution of adiposity phenotypes in the risk of hyperuricemia among Chinese rural population. Clin Rheumatol. (2018) 37:2221–31. doi: 10.1007/s10067-018-4143-x 29770928

[18] Zou J Xiong H Zhang H Hu C Lu S Zou Y. Association between the cardiometabolic index and non-alcoholic fatty liver disease: insights from a general population. BMC Gastroenterol. (2022) 22:20. doi: 10.1186/s12876-022-02099-y 35021995 PMC8756663

[19] Kahn HS. The “lipid accumulation product” performs better than the body mass index for recognizing cardiovascular risk: a population-based comparison. BMC Cardiovasc Disord. (2005) 5:26. doi: 10.1186/1471-2261-5-26 16150143 PMC1236917

[20] Eddowes PJ Sasso M Allison M Tsochatzis E Anstee QM Sheridan D. Accuracy of fibroScan controlled attenuation parameter and liver stiffness measurement in assessing steatosis and fibrosis in patients with nonalcoholic fatty liver disease. Gastroenterology. (2019) 156:1717–30. doi: 10.1053/j.gastro.2019.01.042 30689971

[21] Chen X Shi F Xiao J Huang F Cheng F Wang L. Associations between abdominal obesity indices and nonalcoholic fatty liver disease: chinese visceral adiposity index. Front Endocrinol (Lausanne). (2022) 13:831960. doi: 10.3389/fendo.2022.831960 35360076 PMC8960385

[22] Ismaiel A Jaaouani A Leucuta DC Popa SL Dumitrascu DL. The visceral adiposity index in non-alcoholic fatty liver disease and liver fibrosis-systematic review and meta-analysis. Biomedicines. (2021) 9(12):1890. doi: 10.3390/biomedicines9121890 34944706 PMC8698356

[23] Li Q Wang L Wu J Wang J Wang Y Zeng X. Role of age, gender and ethnicity in the association between visceral adiposity index and non-alcoholic fatty liver disease among US adults (NHANES 2003-2018): cross-sectional study. BMJ Open. (2022) 12:e058517. doi: 10.1136/bmjopen-2021-058517 PMC8938699 35314476

[24] Liu Y Wang W. Sex-specific contribution of lipid accumulation product and cardiometabolic index in the identification of nonalcoholic fatty liver disease among Chinese adults. Lipids Health Dis. (2022) 21:8. doi: 10.1186/s12944-021-01617-3 35027066 PMC8759215

[25] Yan L Hu X Wu S Cui C Zhao S. Association between the cardiometabolic index and NAFLD and fibrosis. Sci Rep. (2024) 14:13194. doi: 10.1038/s41598-024-64034-3 38851771 PMC11162484

[26] Ebrahimi M Seyedi SA Nabipoorashrafi SA Rabizadeh S Sarzaeim M Yadegar A. Lipid accumulation product (LAP) index for the diagnosis of nonalcoholic fatty liver disease (NAFLD): a systematic review and meta-analysis. Lipids Health Dis. (2023) 22:41. doi: 10.1186/s12944-023-01802-6 36922815 PMC10015691

[27] Nielsen S Guo Z Johnson CM Hensrud DD Jensen MD. Splanchnic lipolysis in human obesity. J Clin Invest. (2004) 113:1582–8. doi: 10.1172/JCI21047 PMC419492 15173884

[28] Milić S Lulić D Štimac D. Non-alcoholic fatty liver disease and obesity: biochemical, metabolic and clinical presentations. World J Gastroenterol. (2014) 20:9330–7. doi: 10.3748/wjg.v20.i28.9330 PMC4110564 25071327

[29] Stefan N Kantartzis K Häring HU. Causes and metabolic consequences of Fatty liver. Endocr Rev. (2008) 29:939–60. doi: 10.1210/er.2008-0009 18723451

[30] Vanni E Bugianesi E Kotronen A De Minicis S Yki-Järvinen H Svegliati-Baroni G. From the metabolic syndrome to NAFLD or vice versa? Dig Liver Dis. (2010) 42:320–30. doi: 10.1016/j.dld.2010.01.016 20207596

[31] Roden M Price TB Perseghin G Petersen KF Rothman DL Cline GW. Mechanism of free fatty acid-induced insulin resistance in humans. J Clin Invest. (1996) 97:2859–65. doi: 10.1172/JCI118742 PMC507380 8675698

[32] Shulman GI. Cellular mechanisms of insulin resistance. J Clin Invest. (2000) 106:171–6. doi: 10.1172/JCI10583 PMC314317 10903330

[33] Donnelly KL Smith CI Schwarzenberg SJ Jessurun J Boldt MD Parks EJ. Sources of fatty acids stored in liver and secreted via lipoproteins in patients with nonalcoholic fatty liver disease. J Clin Invest. (2005) 115:1343–51. doi: 10.1172/JCI23621 PMC1087172 15864352

[34] Yu X Hao M Liu Y Ma X Lin W Xu Q. Liraglutide ameliorates non-alcoholic steatohepatitis by inhibiting NLRP3 inflammasome and pyroptosis activation via mitophagy. Eur J Pharmacol. (2019) 864:172715. doi: 10.1016/j.ejphar.2019.172715 31593687

[35] Guilherme A Virbasius JV Puri V Czech MP. Adipocyte dysfunctions linking obesity to insulin resistance and type 2 diabetes. Nat Rev Mol Cell Biol. (2008) 9:367–77. doi: 10.1038/nrm2391 PMC2886982 18401346

[36] Fontana L Eagon JC Trujillo ME Scherer PE Klein S. Visceral fat adipokine secretion is associated with systemic inflammation in obese humans. Diabetes. (2007) 56:1010–3. doi: 10.2337/db06-1656 17287468

[37] Park HS Park JY Yu R. Relationship of obesity and visceral adiposity with serum concentrations of CRP, TNF-alpha and IL-6. Diabetes Res Clin Pract. (2005) 69:29–35. doi: 10.1016/j.diabres.2004.11.007 15955385

[38] Kintscher U Hartge M Hess K Foryst-Ludwig A Clemenz M Wabitsch M. T-lymphocyte infiltration in visceral adipose tissue: a primary event in adipose tissue inflammation and the development of obesity-mediated insulin resistance. Arterioscler Thromb Vasc Biol. (2008) 28:1304–10. doi: 10.1161/ATVBAHA.108.165100 18420999

[39] Sutti S Jindal A Locatelli I Vacchiano M Gigliotti L Bozzola C. Adaptive immune responses triggered by oxidative stress contribute to hepatic inflammation in NASH. Hepatology. (2014) 59:886–97. doi: 10.1002/hep.26749 24115128

[40] Ozercan AM Ozkan H. Vibration-controlled transient elastography in NAFLD: review study. Euroasian J Hepatogastroenterol. (2022) 12:S41–s5. doi: 10.5005/jp-journals-10018-1365 PMC9681576 36466098

[41] Younossi ZM Loomba R Anstee QM Rinella ME Bugianesi E Marchesini G. Diagnostic modalities for nonalcoholic fatty liver disease, nonalcoholic steatohepatitis, and associated fibrosis. Hepatology. (2018) 68:349–60. doi: 10.1002/hep.29721 PMC6511364 29222917

